# Phosphoglyceride crystal deposition disease in the abdominal wall: a case report

**DOI:** 10.1186/s40792-018-0516-2

**Published:** 2018-09-19

**Authors:** Osamu Nakahara, Hideo Baba

**Affiliations:** 1 Department of Surgery, Taragi Municipal Hospital, 4210 Taragi, Taragi-machi, Kuma-gun, Kumamoto, 868-0598 Japan; 2 Department of Gastroenterological Surgery, Graduate School of Medical Sciences, Kumamoto University, 1-1-1 Honjo, Chuo-ku, Kumamoto City, Kumamoto, 860-8556 Japan

**Keywords:** Soft tissue tumor, Phosphoglyceride crystal deposition disease, Foreign-body granuloma

## Abstract

**Background:**

Phosphoglyceride crystal deposition disease (PGDD) is characterized by phosphoglyceride crystal deposition that simulates neoplasia in soft tissue scars or bone. Reports of PGDDs are rare. Here, we present the case of a patient with PGDD in the abdominal wall.

**Case presentation:**

A 57-year-old Japanese man with worsening right lower abdominal pain had no significant family or occupational history. Laboratory data showed elevated inflammatory markers with a white blood cell count of 14,400 × 10^9^/L and C-reactive protein of 11.8 mg/L, but no other abnormalities. Helical computed tomography (CT) revealed a tumor in the abdominal wall (longest dimension, approximately 10 cm). Positron emission tomography–CT revealed fluorodeoxyglucose accumulation in the mass only (SUVmax, 41). Clinical and radiographic findings suggested malignant lymphoma, undifferentiated sarcoma, or liposarcoma. He underwent exploratory laparotomy and further treatment. At surgery, we found a huge milky-whitish mass with a rough surface in the transversus abdominis. Complete resection was performed and his postoperative recovery was good. Surprisingly, the final pathologic diagnosis was phosphoglyceride crystal deposition disease with the characteristic crystal deposition in a corolla shape, histiocytic reaction with abundant foreign-body-type giant cells, and no evidence of neoplasia. The patient remains asymptomatic with no disease recurrence.

**Conclusion:**

Although phosphoglyceride crystal deposition disease in the abdominal wall is rarely encountered in clinical practice, its inclusion in differential diagnosis is important. Given the occurrence at sites of invasive procedures, we believe efforts to reduce invasiveness when performing surgery and follow-up for early detection of recurrence are important.

## Background

Phosphoglyceride crystal deposition disease (PGDD) is characterized by phosphoglyceride crystal deposition that simulates neoplasia in soft tissue scars or bone. The condition is rarely encountered in the clinical setting and is sometimes strongly suspected of being a malignant tumor based on clinical and radiographic findings. The lesions often occur over injection sites or surgical scars, such as the periumbilical area or upper arm, suggesting a possible relationship with these procedures.

In this report, we describe a case of PGDD in the abdominal wall, which was resected surgically after being initially diagnosed as a malignant tumor.

## Case presentation

A 57-year-old Japanese man was referred to our hospital because of increasing right lower abdominal pain. He had a history of appendectomy at the age of 17 years with no significant family or occupational history.

**Fig. 1:**
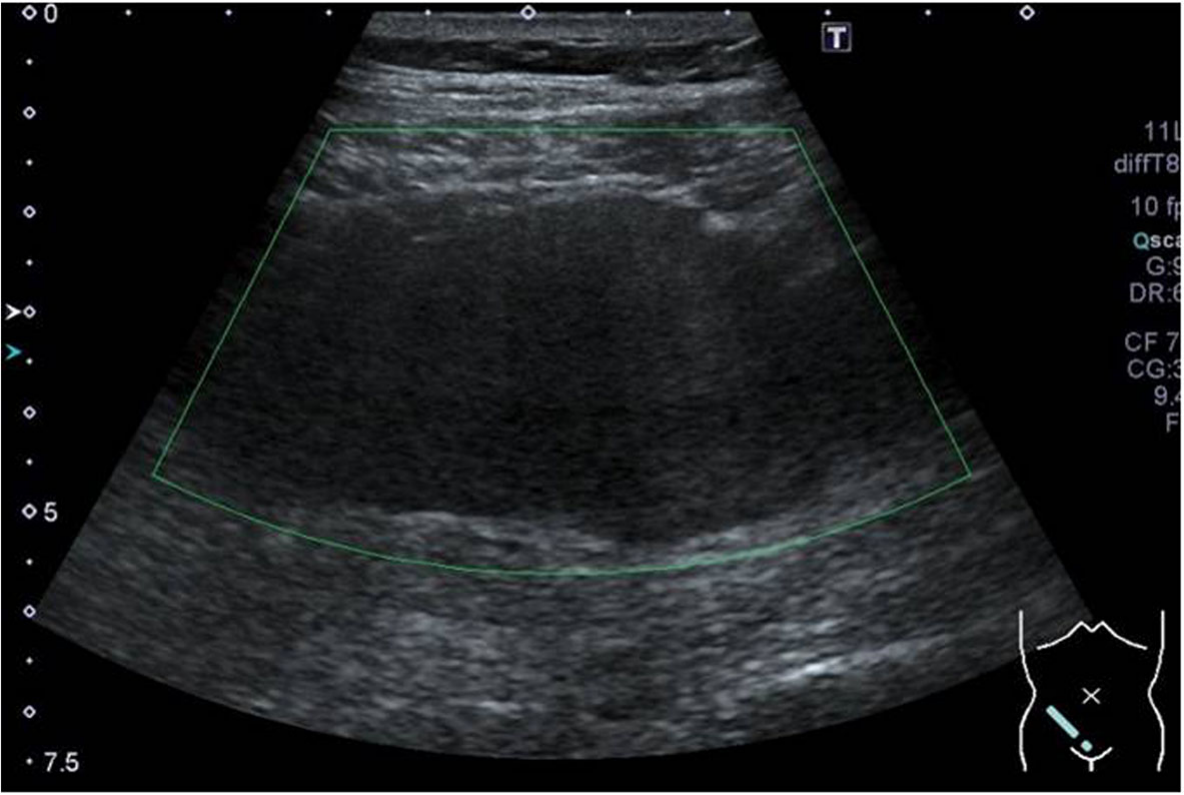
Transabdominal ultrasonographic image of a tumor measuring 10 cm in diameter showing a homogeneous echo pattern

**Fig. 2:**
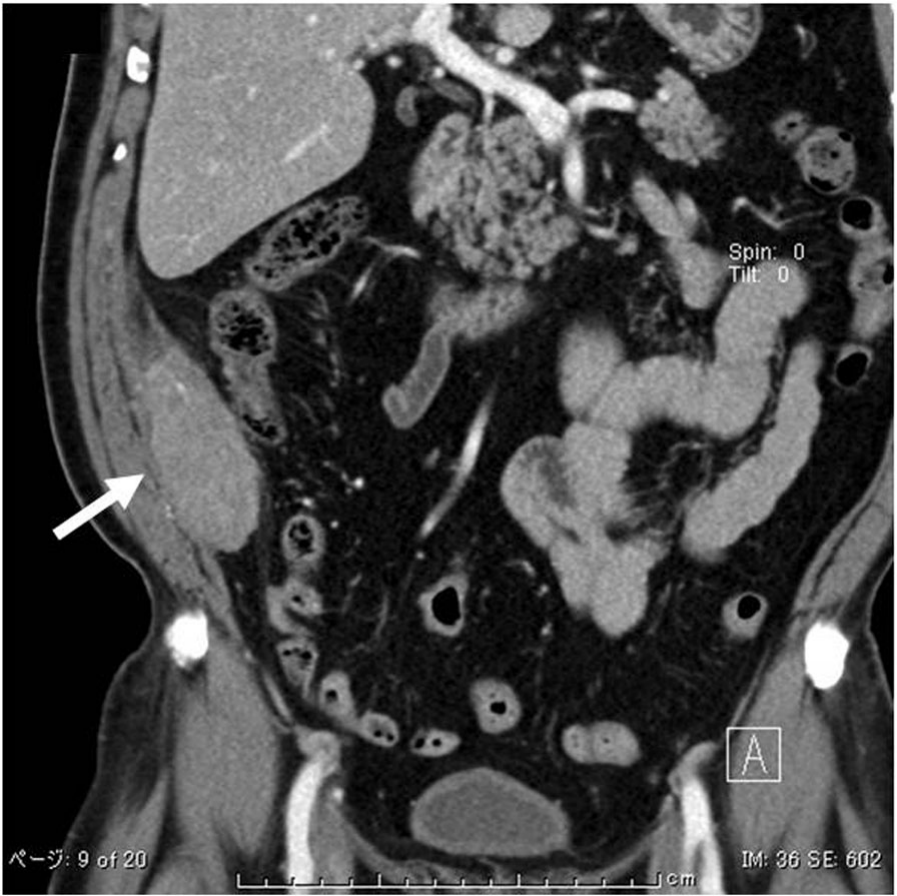
Helical computed tomography (CT) indicating a solid mass in the abdominal wall (arrow)

**Fig. 3:**
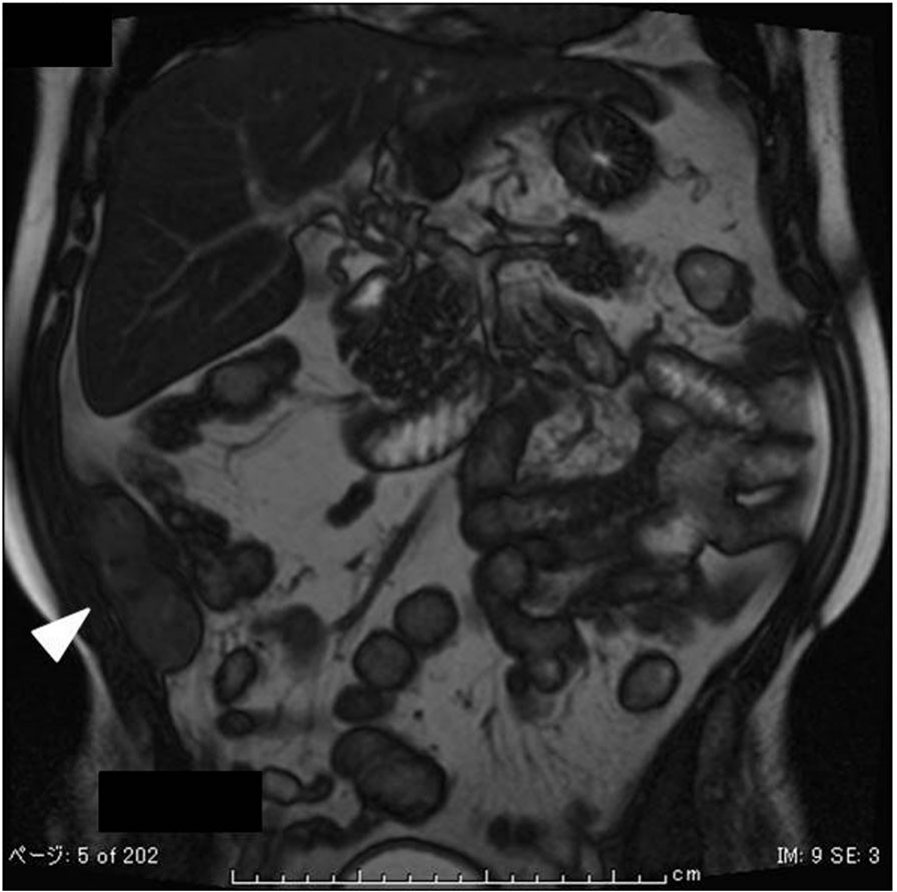
Magnetic resonance imaging of the abdomen showing elliptical masses in the transverse abdominal wall exhibiting low intensity on T2-weighted images (arrowhead)

**Fig. 4:**
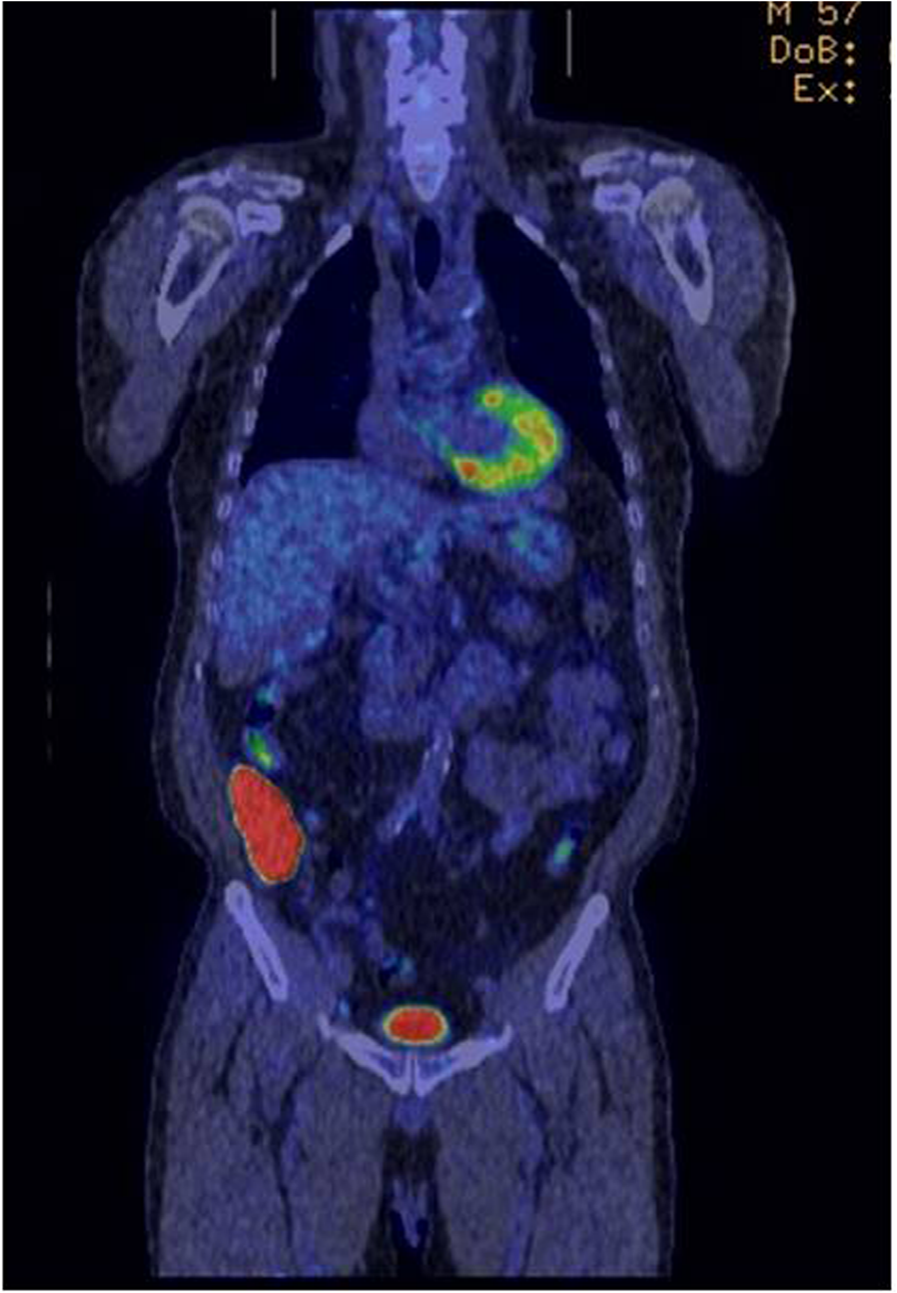
FDG-PET showing abnormal accumulation (SUVmax 41) in the abdominal wall

On initial examination, an abdominal wall tumor (largest dimension, approximately 10 cm in diameter) was detected using transabdominal ultrasound (Fig. 1). Laboratory data revealed elevated inflammatory markers (WBC = 14,400 × 10^9^/L, CRP = 11.8 mg/L); major tumor markers (carcinoembryonic antigen, CA19-9, and soluble IL-2 receptor) were within normal limits. Helical computed tomography (CT) also revealed a solid mass (largest dimension, 10 cm in diameter) in the abdominal wall (Fig. 2). Magnetic resonance imaging showed a mass that exhibited low intensity on T2-weighted images, slightly high intensity on diffusion-weighted images, and gradual reinforcement on dynamic study (Fig. 3). Positron emission tomography–CT revealed fluorodeoxyglucose accumulation in the mass only (SUVmax, 41) (Fig. 4). Because clinical and radiographic findings suggested malignant lymphoma, undifferentiated sarcoma, or liposarcoma, he underwent exploratory laparotomy and treatment.

**Fig. 5:**
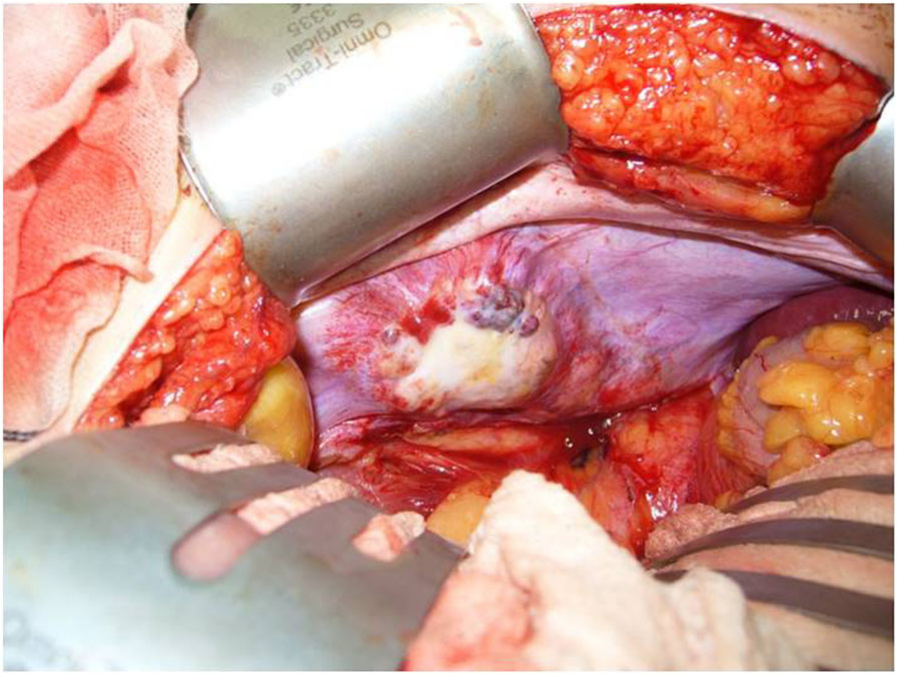
Intraoperative abdominal view of an elastic, hard, milky-white tumor with a rough surface. Capillary development is evident in the right lower quadrant

Intraoperative findings at laparotomy were an elastic, hard, milky-whitish mass with a rough surface and capillary growth in the right lower quadrant (Fig. 5). On inspection and palpation, a malignant tumor was strongly suspected. No other tumor suspicious of a primary lesion was found in the intraabdominal organs, including the gastrointestinal tract. The mass was completely removed, and the surgical margin secured. The total weight of the mass was 120 g. No complications were observed during the perioperative period, and the patient was discharged on postoperative day 7.

**Fig. 6:**
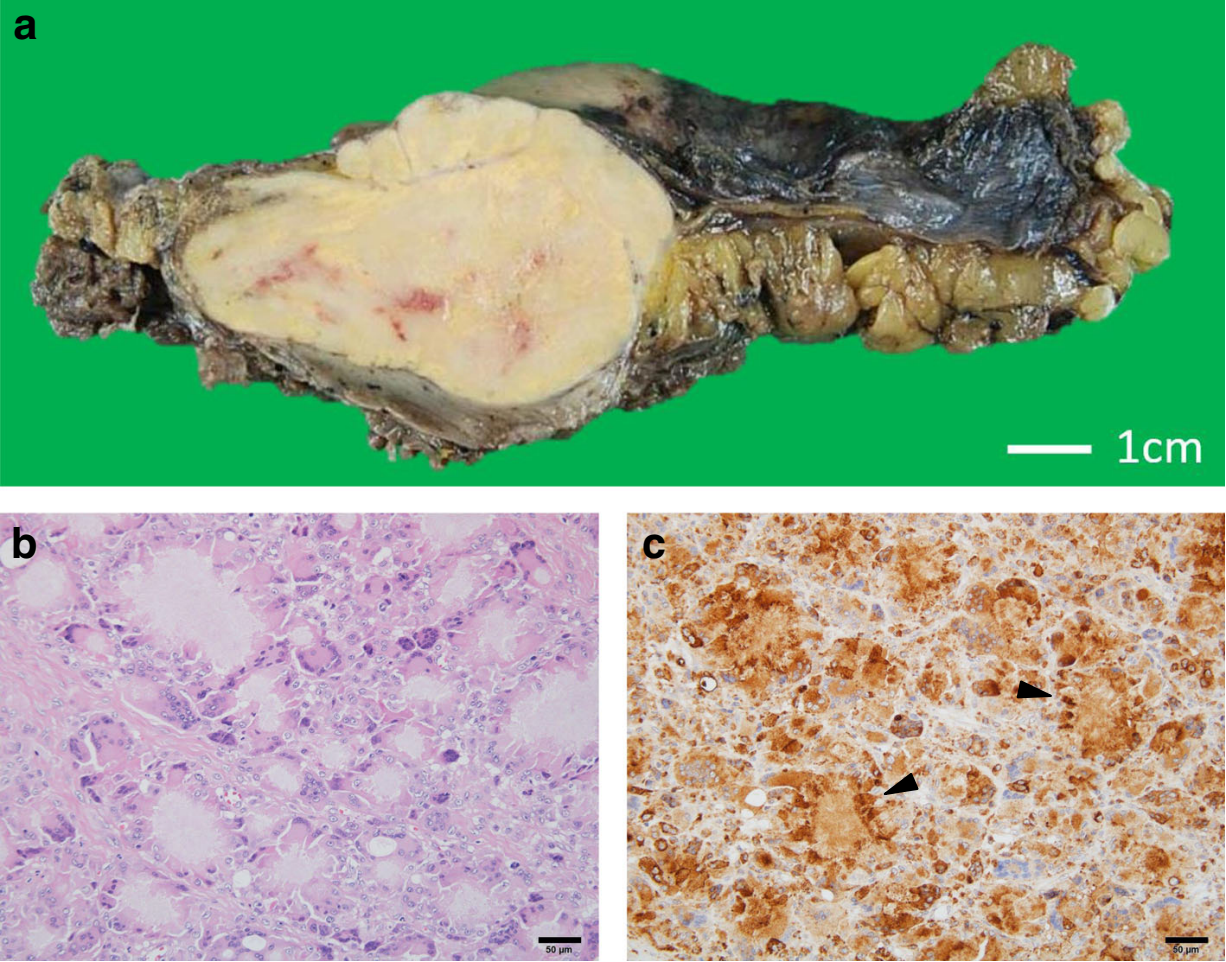
**a** Macroscopic findings of the cut surface of the tumoral mass showing a lobulated, firm, yellowish-white portion. **b** Histological findings. Hematoxylin and eosin staining showing a foreign-body granuloma consisting of string-like crystals and a foreign-body giant cell. Many tiny granulomas with central bluish-pink crystals surrounded by macrophages are evident. Bar 50 μm. **c** Immunohistological staining using anti-CD68 antibody (clone KP1) against CD68, a known macrophage surface marker, was positive in cells surrounding phosphoglyceride crystals (arrowhead) bar 50 μm.

Pathologic examination revealed that these masses were foreign-body granulomas consisting of string-like crystals and a foreign-body giant cell (Fig. 6a, b). Immunohistological staining using anti-CD68 antibody (clone KP1) against the CD68 antigen, which is a known macrophage surface marker, was positive in cells surrounding phosphoglyceride crystals (Fig. 6c).

The patient underwent regular follow-up abdominal ultrasound examination and CT postoperatively. As of 3 years after the procedure, no signs of recurrence have been detected.

PGDD is a rare disease characterized by deposition of phosphoglyceride crystals, often simulating neoplasm in a scar of soft tissue or bone. It can sometimes form a large mass and be misdiagnosed as a malignant tumor.

Little is known about its etiology and pathogenesis. This deposition disease is apparently triggered by injury and subsequent macrophage aggregation, with a histological picture of deposited crystal radiating from the cell membranes of epithelioid cells. The macrophages themselves may contribute to the formation of a nidus for the crystals. One hypothesis is that localized disturbance of phosphoglyceride metabolism within the macrophages may be initiated by local inflammation, leading to progressive amplification of macrophage infiltration and crystal deposition.

To our knowledge, only 10 cases of PGDD have been reported previously, including our case. The available clinical information on these cases is summarized in Table 1 [1–7]. The mean age of patients is 57 (range, 37–76) years. There appears to be no gender predilection. No congenital abnormalities or family history of metabolic disorders have been found. Deposition was characteristically noted at intramuscular injection sites or postoperative sites like the gluteal muscles and the deltoid, abdominal wall soft tissue, scapula, spine, myocardium, and pelvic soft tissue. The size of the tumors reported is highly variable, from 3.5 cm to the size of an infant’s head, but in general it is a relatively large tumor with a mean size of 8.9 cm. The time from the initial invasion to confirmation of the tumor was at least 20 years and was 45 years in the longest case. About half of the cases had multiple lesions. In most cases, excisional surgery was performed due to local tumor formation without apparent symptoms of inflammation. Tumors that occurred at postoperative sites were suspected to be true malignant neoplasms or recurrent tumors. In one case, intraoperative rapid pathological diagnosis revealed PGDD, so complete resection was not performed and the patient was observed clinically.

**Table 1:** Clinical summary of phosphoglyceride crystal deposition disease

Age	Gender	Location	Past medical history	Interval period	No. of tumors	Tumor size	Reference
58	M	Buttock muscle	Not detected	Not detected	Single	11 cm	Kubo, 1992 [1]
62	F	Oral soft tissue	Appendectomy, dental treatment	20 years over	Multiple	11 cm	Miura, 2000 [2]
		Abdominal soft region					
		Brachial muscle					
51	M	Abdominal soft region	Post-gastrectomy	33 year	Multiple	3.5 cm	Yachida, 2002 [3]
58	M	Abdominal soft region	Post-gastrectomy	40 years over	Single	Infant head size	Miura, 2004 [4]
64	F	Abdominal soft region	Post-gastrectomy	35 year	Single	4 cm	Miura, 2004
64	F	Scapular bone	Not detected	Not detected	Single	10 cm	Miura, 2004
76	F	Spine	Lumbar anesthesia	45 year	Multiple	Not detected	Nishimura, 2005 [5]
37	M	Anterior mediastinum	Ventricular septal defect	35 year	Single	6 cm	Shoji, 2007 [6]
50	F	Pelvic soft tissues	Cesarean delivery	26 year	Multiple	10 cm	Yamada, 2015 [7]
57	M	Abdominal soft region	Appendectomy	40 year	Single	10 cm	Our case

No specific markers exist for PGDD, and differentiating this disease from malignant tumors without pathological examination is difficult. We considered needle biopsy in the present case but decided against it given the risk of needle tract seeding if malignant.

In the present case, exploratory laparotomy, not laparoscopic surgery, was performed for a suspected huge malignant tumor of the abdominal wall, based on radiographic examination. Our patient had a history of appendectomy at the age of 17 years, and local inflammation at the operation site was suspected to have led to progressive amplification of macrophage infiltration and crystal deposition.

Postoperative follow-up has not revealed any findings suspicious of recurrence in our case. However, there are reports in the literature of recurrence at 4 years after surgery [7]. Few studies have reported on the process of crystallization and deposition of phosphoglycerides and so the mechanism remains unclear. Local injection of a bovine-derived medication has been suggested as causative, yet that alone does not explain PGDD, including the present case. In reviewing previous reports, a few cases suggest that during the process of phosphoglyceride metabolism, a locally invasive procedure increases macrophage activation and leads to phosphoglyceride deposition. Moreover, knowledge of the tendency of PGDD to form at sites of invasion may allow a clinician to clinically observe a similar mass found during routine follow-up, and debulking surgery may be considered as a therapeutic option for symptomatic cases.

## Conclusions

PGDD is extremely rare but should be considered in the differential diagnosis of abdominal tumors in patients with a history of abdominal surgery. Because PGDD results from inflammatory response at sites of locally invasive procedures, multiple lesions and recurrence are possible and careful observation of the clinical course is therefore important.

## Abbreviations

CT: Computed tomography
PGDD: Phosphoglyceride crystal deposition disease
